# Deviation of Zipf's and Heaps' Laws in Human Languages with Limited Dictionary Sizes

**DOI:** 10.1038/srep01082

**Published:** 2013-01-30

**Authors:** Linyuan Lü, Zi-Ke Zhang, Tao Zhou

**Affiliations:** 1 Institute of Information Economy, Hangzhou Normal University, Hangzhou 310036, People's Republic of China; 2 Department of Physics, University of Fribourg, Chemin du Musée 3, Fribourg 1700, Switzerland; 3 Beijing Computational Science Research Center, Beijing 100084, People's Republic of China; 4 Web Sciences Center, University of Electronic Science and Technology of China, Chengdu 610054, People's Republic of China

## Abstract

Zipf's law on word frequency and Heaps' law on the growth of distinct words are observed in Indo-European language family, but it does not hold for languages like Chinese, Japanese and Korean. These languages consist of characters, and are of very limited dictionary sizes. Extensive experiments show that: (i) The character frequency distribution follows a power law with exponent close to one, at which the corresponding Zipf's exponent diverges. Indeed, the character frequency decays exponentially in the Zipf's plot. (ii) The number of distinct characters grows with the text length in three stages: It grows linearly in the beginning, then turns to a logarithmical form, and eventually saturates. A theoretical model for writing process is proposed, which embodies the rich-get-richer mechanism and the effects of limited dictionary size. Experiments, simulations and analytical solutions agree well with each other. This work refines the understanding about Zipf's and Heaps' laws in human language systems.

Uncovering the statistics and dynamics of human languages helps in characterizing the universality, specificity and evolution of cultures^1^,^2^,^3^,^4^,^5^,^6^,^7^,^8^,^9^,^10^,^11^,^12^. Two scaling relations, Zipf's law^13^ and Heaps' law^14^, have attracted much attention from academic community. Denote *r* the rank of a word according to its frequency *Z*(*r*), Zipf's law is the relation *Z*(*r*) ~ *r*^−*α*^, with *α* being the Zipf's exponent. Zipf's law was observed in many languages, including English, French, Spanish, Italian, and so on^13^,^15^,^16^. Heaps' law is formulated as *N_t_* ~ *t^λ^*, where *N_t_* is the number of distinct words when the text length is *t*, and *λ* ≤ 1 is the so-called Heaps' exponent. These two laws coexist in many language systems. Gelbukh and Sidorov^17^ observed these two laws in English, Russian and Spanish texts, with different exponents depending on languages. Similar results were recently reported for the corpus of web texts^18^, including the *Industry Sector Database*, the *Open Directory* and the *English Wikipedia*. The occurrences of tags in online resources^19^,^20^, keywords in scientific publications^21^ and words in web pages resulted from web searching^22^ also simultaneously display the Zipf's and Heaps' laws. Interestingly, even the identifiers in programs by Java, C++ and C languages exhibit the same scaling laws^23^.

The Zipf's law in language systems can result from a rich-get-richer mechanism as suggested by the Yule-Simon model^24^,^25^, where a new word is added to the text with probability *q* and an appeared word is randomly chosen and copied with probability 1 − *q*. A word appearing more frequently thus has higher probability to be copied, leading to a power-law word frequency distribution *p*(*k*) ~ *k*^−*β*^, where *k* denotes the frequency and *β* = 1 + 1/(1 − *q*). Dorogovtsev and Mendes modeled the language processing as the evolution of a word web with preferential attachment^26^. Zanette and Montemurro^27^ as well as Cattuto *et al.*^28^ considered the memory effects, namely the recently used words have higher probability to be chosen than the words appeared long time ago. These works can be considered as variants of the Yule-Simon model. Meanwhile, the Heaps' law may originate from the memory and bursty nature of human languages^29^,^30^,^31^.

Recent studies revealed more complicated statistical features of language systems. Wang *et al.*^32^ analyzed representative publications in Chinese, and showed that the character frequency distribution decays exponentially in the Zipf's plot. Lü *et al.*^33^ pointed out that in a growing system, if the appearing frequencies of elements obey the Zipf's law with a stable exponent, then the number of distinct elements grows in a complicated way where the Heaps' law is only an asymptotical approximation. This deviation from the Heaps' law was proved mathematically by Eliazar^34^. Empirical analyses on real language systems showed similar deviations^35^.

Via extensive analysis on Chinese, Japanese and Korean books, we found even more complicated phenomena: (i) The character frequency distribution follows a power law with exponent close to one, at which the corresponding Zipf's exponent diverges. Indeed, the character frequency decays exponentially in the Zipf's plot. (ii) The number of distinct characters grows with the text length in three stages: It grows linearly in the beginning, then turns to a logarithmical form, and eventually saturates. All these unreported phenomena result from the combination of the rich-get-richer mechanism and the limited dictionary sizes, which is verified by a theoretical model.

## Results

### Experiments

We first show some statistical regularities on Chinese, Japanese and Korean literatures, which are representative languages with very limited dictionary sizes if we look at the character level. There are only around 4000 characters being frequently used in Chinese texts (4808, 4759 and 3500 frequently used characters are identified in Taiwan, Hong Kong, and mainland China, respectively), and the number of Japanese and Korean characters are even smaller. Note that, a Korean character is indeed a single syllable consisting of 2–4 letters. We use the term character for convenience and consistence, while one should be aware of the fact that the Korean characters are totally different from Chinese characters: the former are phonographies while the latter are ideographies.

**Figure 1:**
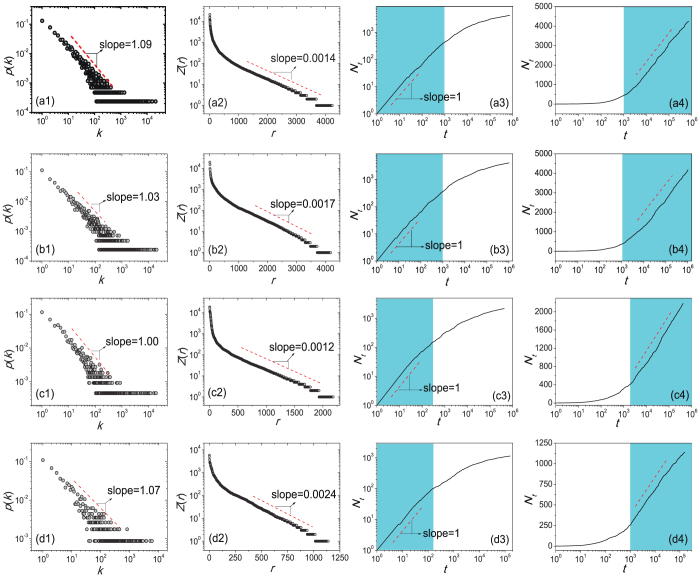
The character frequency distribution of *The Story of the Stone:* (a1) *p*(*k*) with log-log scale and (a2) *Z*(*r*) with log-linear scale. The number of distinct characters versus the text length of *The Story of the Stonein* (a3) log-log scale and (a4) linear-log scale. Similar plots in (b1–b4), (c1–c4) and (d1–d4) are for the books *The Battle Wizard*, *Into the White Night* and *The History of the Three Kingdoms*, respectively. The power-law exponent β is obtained by using the maximum likelihood estimation^36^,^37^, while the exponent in the Zipf's plot is obtained by the least square method excluding the head (the majority of characters in the head play the similar role to the auxiliary words, conjunctions or prepositions in English). We fit the data *r* > 500 for Chinese books and *r* > 200 for Japanese and Korean books.

We start with four famous books, the first two are in Chinese, the third one is in Japanese and the last one is in Korean (see data description in **Methods and Materials**). Figure 1 reports the character frequency distribution *p*(*k*), the Zipf's plot on character frequency *Z*(*r*) and the growth of the number of distinct characters *N_t_* versus the text length *t*. As shown in figure 1, the character frequency distributions are power-law, meanwhile the frequency decays exponentially in the Zipf's plot, which is in conflict with the common sense that a power-law probability density function always corresponds to a power-law decay in the Zipf's plot. Actually, for a power-law probability density distribution *p*(*k*) ~ *k*^−*β*^, usually, a power-law decay can be observed in its corresponding Zipf's plot, say *Z*(*r*) ~ *r*^−*α*^. There exists a relation between two exponents *α* and *β* as 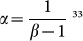 33, and when *β* gets close to 1, the exponent *α* diverges. Under such case, we could not say the corresponding Zipfs distribution is power-law. In principle, the Zipfs distribution can be exponential or in other forms. Therefore, if we observe a nonpower-law decaying in the Zipf's plot, we cannot immediately deduce that the corresponding probability density function is not a power law – it is possibly a power law with exponent close to 1.

**Figure 2:**
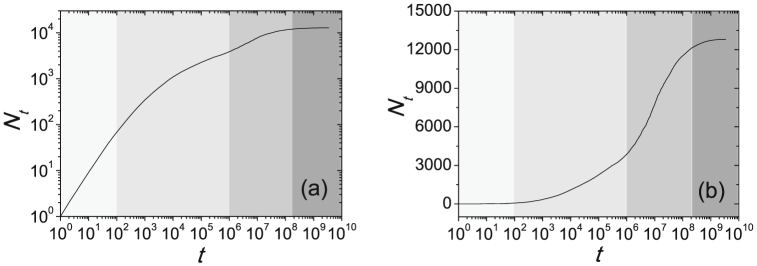
The growth of distinct characters in the corpus of 57755 Chinese books.

Figure 1 also indicates that the growth of distinct characters cannot be described by the Heaps' law. Indeed, there are two distinguishable stages: In the early stage, *N_t_* grows approximately linearly with the text length *t*, and in the later stage, *N_t_* grows logarithmically with *t*. Figure 2 presents the growth of distinct characters for a large collection of 57755 Chinese books consisting of about 3.4 × 10^9^ characters and 12800 distinct characters. In addition to those observed in figure 1, *N_t_* displays a strongly saturated behavior when the text length *t* is much larger than the dictionary size. In summary, the experiments on Chinese, Japanese and Korean literatures show us some novel phenomena: the character frequency obeys a power law with exponent close to 1 while it decays exponentially in the Zipf's plot, and the number of distinct characters grows in three distinguishable stages (figure 2 also shows the crossover between linear growth and logarithmic growth).

### Model

**Figure 3:**
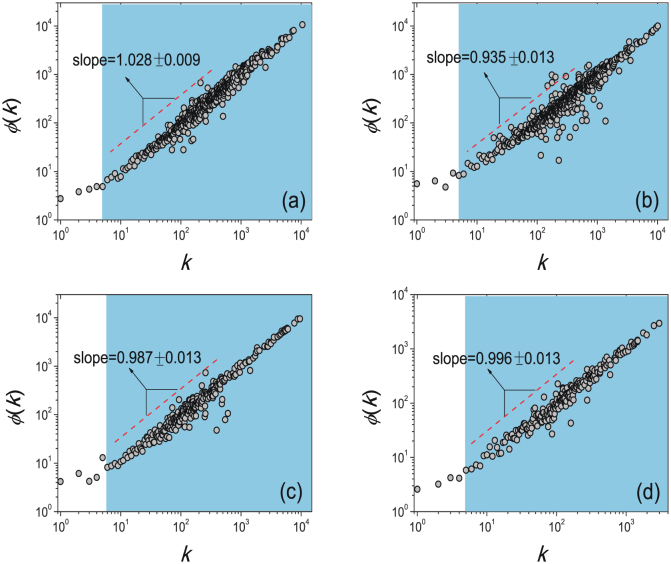
*φ*(*k*) versus *k* in a log-log scale for the four representative books.

Text generation was usually described as a rich-get-richer process like the aforementioned Yule-Simon model^24^. Before establishing the model, we first test whether the rich-get-richer mechanism works for writing process. We denote *φ*(*k*) the average probability that a character appeared *k* times will appear again (see **Methods and Materials** how to measure *φ*(*k*)). As shown in figure 3, *φ*(*k*) ~ *k^γ^* for all the four books with *γ* ≈ 1, indicating a linearly rich-get-richer effect like the preferential attachment in evolving scale-free networks^38^.

In the model, we consider a language with finite dictionary size, *V*, of distinct characters. At each time step, one character in the dictionary will be selected to form the text. Motivated by the rich-getricher mechanism, at time step *t* + 1, if the character *i* has been used *k_i_* times, it will be selected with the probability proportional to *k_i_* (according to the approximately linear relation between *φ*(*k*) and *k*), as 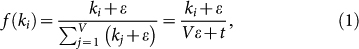
where *ε* is the initial attractiveness of each character (*ε* > 0 ensures that every character has chance to be selected).

**Figure 4:**
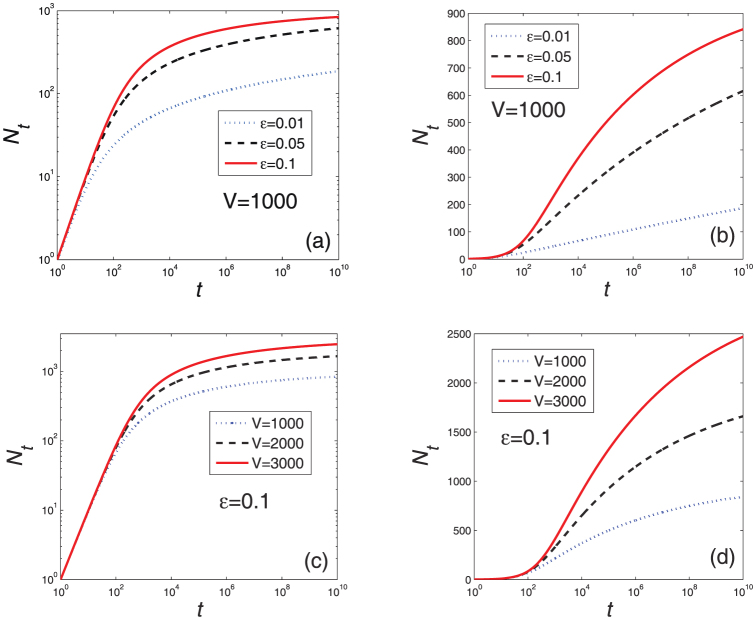
Growth of the number of distinct characters versus time for different *V* and *ε* according to Eq. 2.

This growing dynamics can be analytically solved as (see **Methods and Materials**) 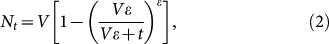
which embodies three stages of growth of *N_t_*: (i) In the very early stage, when *t* is much smaller than *Vε*, 
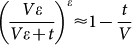
 and thus *N_t_* ≈ *t*, corresponding to a short period of linear growth. (ii) When *t* is of the same order of *Vε*, if *ε* is very small, *N_t_* could be much smaller than *V*. Expanding 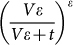 by Taylor series as 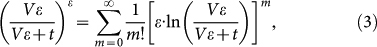
and neglecting the high-order terms (*m* ≥ 2) under the condition *ε* ≪ 1, one can obtain a logarithmical solution 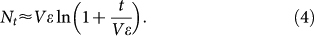
As indicated in figure 2, there is a crossover between the first two stages. (iii) When *t* gets larger and larger, *N_t_* will approach to *V* and thus both 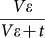 and 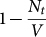 are very small, leading to a very slow growing of *N_t_* according to Eq. 7 (see **Methods and Materials**). These three stages predicted by the analytical solution are in good accordance with the above empirical observations (see figure 2). Figure 4 reports the numerical results on Eq. 2. Agreeing with the analysis, when *t* is small, *N_t_* grows in a linear form as shown in Fig. 4(a) and 4(c), and in Fig. 4(b) and 4(d), the linear part in the middle region indicates a logarithmical growth as predicted by Eq. 4.

According to the master equation (see **Methods and Materials**), the character frequency distribution can be analytically solved as 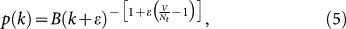
where *B* is the normalization factor. The result shows that the character frequency follows a power-law distribution with exponent varying in time. Considering the finite dictionary size, in the large limit of *t*, *N_t_* → *V* and thus the power-law exponent, 
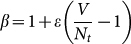
, approaches one. The corresponding frequency-rank relation in the Zipf's plot is (see **Methods and Materials**) 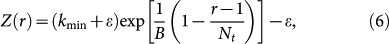
where *k*_min_ = *Z*(*N_t_*) is the smallest frequency. In a word, this simple model accounting for the finite dictionary size results in a power-law character frequency distribution *p*(*k*) ~ *k*^−*β*^ with exponent *β* close to 1 and an exponential decay of *Z*(*r*), perfectly agreeing with the empirical observations on Chinese, Japanese and Korean books.

**Figure 5:**
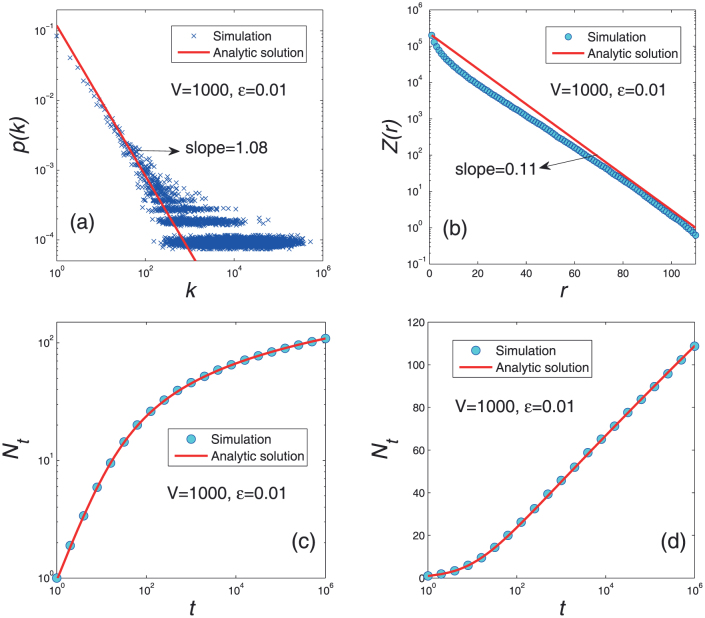
Comparison between simulations results (blue data points) and analytical solutions (red curves) for typical parameters *V* = 1000 and *ε* = 0.01.

Figure 5 reports the simulation results. The power-law frequency distribution, the exponential decay of frequency in the Zipf's plot and the linear to logarithmic transition in the growth of the distinct number of characters are all observed and in good accordance with the analytical solutions.

**Figure 6:**
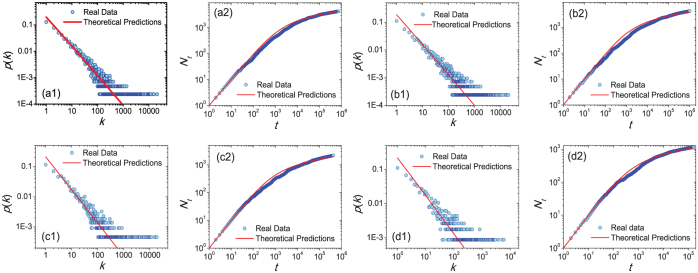
Comparison between analytical predictions (red lines) and the real data (blue circles) on probability density functions (a1, b1, c1, d1) and the growing process of the number of distinct characters (a2, b2, c2, d2).

Figure 6 directly compares the analytical predictions and the real data. They agree with each other quantitatively. In comparison, predictions from known models are qualitatively different from the present observations. For example, in the Yule-Simon model^24^, the predicted power-law exponent is larger than 2 and the number of distinct characters grows linearly with the text size. In the Yule-Simon model with memory^28^, the growth of distinct words follows a linear process and the word frequency distribution is not a power-law. Dorogovtsev and Mendes^26^ proposed a word-web-based model, where the growth of distinct words follows the Heaps law with exponent 0.5 and the power-law exponent can be either 1.5 or 3, also far different from the current results.

## Discussion

Previous statistical analyses about human languages overwhelmingly concentrated on Indo-European family, where each language consists of a huge number of words. In contrast, languages consisting of characters, though cover more than a billion people, received less attention. These languages include Chinese, Japanese, Korean, Vietnamese, Jurchen language, Khitan language, Makhi language, Tangut language, and many others. Significant differences between these two kinds of languages lie in many aspects. Taking English and Chinese as examples. Firstly, the number of words in English is more than 100 times larger than the number of characters in Chinese. Secondly, no dictionary contains all possible words in English. Basically, everyone could create some new words. New words may result from new techniques, new biological species, or new names. Old words connected by - is also counted as a new one. Instead, generally we cannot give birth to a new Chinese character. Therefore, for English text, absolute saturation cannot appear since it is very possible to find a piece of new words even after a large collection of English literatures. Thirdly, the number of words in English grows very quickly. The *Encyclopedia Americana* (Volume 10, Grolier, 1999) said “The vocabulary has grown from 50000 to 60000 words in Old English to the tremendous number of entries − 650000 to 750000 − in an unabridged dictionary of today. In December 2010 a joint Harvard/Google study found the language to contain 1022000 words and to expand at the rate of 8500 words per year. In contrast, the number of characters in Chinese decreases from 47035 characters in 1716 (the 42-volume Chinese dictionary compiled during the reign of *Emperor Kang Xi* in the *Qing Dynasty*) to about 8000 characters in 1953 according to the *New Chinese Dictionary*. Therefore, in the future, we are not expected to see the saturation of distinct English words either.

The above-mentioned distinctions lead to remarkably different statistical regularities between character-formed languages and word-formed languages. Newly reported features for character-formed languages include an exponential decay of character frequency in the Zipf's plot associated with a power-law frequency distribution with exponent close to 1, and a multi-stage growth of the number of distinct characters. These findings not only complement our understanding of scaling laws in human languages, but also refine the knowledge about the relationship between the power law and the Zipf's law, as well as the applicability of the Heaps' law. As a result, we should be careful when applying the Zipf's plot for a power-law distribution with exponent around 1, such as the cluster size distribution in two-dimensional self-organized critical systems^39^, the inter-event time distribution in human activities^40^, the family name distribution^41^, the species lifetime distribution^42^, and so on. Meanwhile, we cannot deny a possibly power-law distribution just from a non-power-law decay in the Zipf's plot^32^.

The currently reported regularities, deviating from the well-known Zipf's and Heaps' laws, can be reproduced by considering finite dictionary size in a rich-get-richer process. Different from the well-known finite-size effects that vanish in the thermodynamic limit, the effects caused by finite dictionary size get stronger as the increasing of the system size. Finite choices must be a general condition in selecting dynamics, but not a necessary ingredient in growing systems. For example, also based on the rich-get-richer mechanism, neither the linear growing model^38^ nor the accelerated growing model^43^ (treating total degree as the text length and nodes as distinct characters, the accelerated networks grow in the Heaps' manner^33^) has considered such ingredient. The present model could distinguish the selecting dynamics from general dynamics for growing systems.

## Methods

### Data description

Four books are analyzed in this article: (i) *The Story of the Stone*, written by Xueqin Cao in the mid-eighteenth century during the reign of *Emperor Chien Lung* in the *Qing Dynasty*; (ii) *The Battle Wizard*, a kungfu novel written by Yong Jin; (iii) *Into the White Night*, a modern novel written by Higashino Keigo; (iv) *The History of the Three Kingdoms*, a very famous history book by Shou Chen in China and then translated into Korean. These books cover disparate topics and types and were accomplished in far different dates. The basic statistics of these books are presented in Table 1. In addition, we investigate a corpus of 57755 Chinese books consisting of about 3.4 × 10^9^ characters and 12800 distinct characters.

### Measuring the strength of rich-get-richer mechanism

Similar to the method measuring the preferential attachment in evolving networks^44^, for each book under investigation, we divide it into two parts: Part I contains a fraction *ρ* of characters appeared early and Part II contains the remain fraction 1 − *ρ* of characters. For each character *i* in Part II, if *i* did not appear in Part I, nothing happens, while if *i* appeared *k* times in Part I, we add one to *φ*′(*k*) whose initial value is zero. Note that, *i* may appear more than once in Part II and thus contribute more than one to *φ*′(*k*). Accordingly, *φ*′(*k*) is the number of characters in Part II that appeared just *k* times in Part I. Dividing *φ*′(*k*) by the number of distinct characters that appeared *k* times in Part I, we obtain *φ*(*k*). We have checked that the results are not sensitive to *ρ* unless *ρ* is too small or too large, therefore we only show the results for *ρ* = 0.5.

### Growing dynamics of distinct characters

Assuming that at time *t*, there are *N_t_* distinct characters in the text. The selection at time *t* + 1 can be equivalently divided into two complementary yet repulsive actions: (i) to select a character from the dictionary with probability proportional to *ε*, or (ii) to select a character from the *N_t_* characters in the created text with probability proportional to its appeared frequency. Therefore the probability to choose a character from the dictionary is 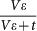, whereas 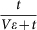 from the created text. A character chosen from the created text is always old, while a character chosen from the dictionary could be new with probability 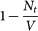. Accordingly, the probability that a new character appears at the *t* + 1 time step, namely the growing rate of *N_t_*, is 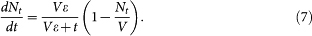
With the boundary conditions *N*_0_ = 0, one can arrive to the solution Eq. 2.

### Character frequency distribution

Denote by *n*(*t*, *k*) the number of distinct characters that appeared *k* times until time *t*, then *n*(*t*, *k*) = *N_t_p*(*k*). According to the master equations, we have 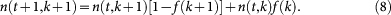
Substituting Eq. 1 into Eq. 8, we obtain 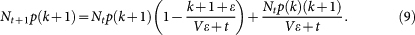
Via continuous approximation, it turns to be the following differential equation 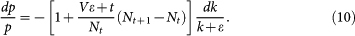
Substituting *N_t_*_+1_ − *N_t_* = *dN_t_*/*dt* and Eq. 7 into Eq. 10, we get the solution 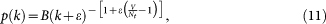
where *B* is the normalization factor. Under the continuous approximation, the cumulative distribution of character frequency can be written as 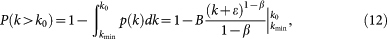
where *k*_min_ is the smallest frequency. When *β* → 1, *k*^1−*β*^ ≈ 1 + (1 − *β*)ln*k*, and thus 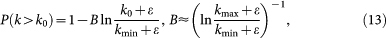
where *B* is obtained by the normalization condition 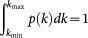 and *k*_max_ is the highest frequency. According to Eq. 13, there are 
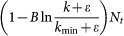
 characters having appeared more than *k* times. That is to say, a character having appeared *k* times will be ranked at 
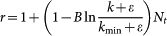
. Therefore 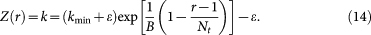


## Author Contributions

Conceived and designed the experiments: LL TZ. Analytical analysis: LL. Performed the experiments: LL ZKZ. Analyzed the data: LL ZKZ TZ. Wrote the paper: LL TZ.

## Figures and Tables

**Table 1:** The basic statistics of the four books. *β* is the exponent of the power-law frequency distribution and *N_T_* is the total number of distinct characters

Language	Book	*T*	*N_T_*	*k_max_*	*k_min_*	*β*
Chinese	The Story of the Stone	727601	4239	21054	1	1.09
Chinese	The Battle Wizard	1020336	4178	20028	1	1.03
Japanese	Into the White Night	420935	2182	18992	1	1.00
Korean	History of the Three Kingdoms	157201	1139	5929	1	1.07

## References

[b1] Hawkins J. A. & Gell-Mann M. The Evolution of Human Languages (Addison-Wesley, Reading, Massachusetts, 1992).

[b2] Caplan D. Language: Structure, Processing and Disorders (MIT Press, Cambidge, 1994).

[b3] Lightfoot D. The Development of Language: Acquisition, Changes and Evolution (Blackwell, Oxford, 1999).

[b4] Nowak M. A. & Krakauer D. C. The evolution of language. Proc. Natl. Acad. Sci. U.S.A. 96, 8028–8033 (1999). 10393942 10.1073/pnas.96.14.8028 PMC22182

[b5] Cancho R. F. i. & Solé R. V. The small world of human language. Proc. R. Soc. Lond. B 268, 2261–2265 (2001). 10.1098/rspb.2001.1800 PMC1088874 11674874

[b6] Nowak M. A., Komarova N. L. & Niyogi P. Computational and evolutionary aspects of language. Nature 417, 611–617 (2002). 12050656 10.1038/nature00771

[b7] Hauser M. D., Chomsky N. & Fitch W. T. The faculty of language: What is it, who has it, and how did it evolve? Science 298, 1569–1579 (2002). 12446899 10.1126/science.298.5598.1569

[b8] Abrams D. & Strogatz S. H. Modelling the dynamics of language death. Nature 424, 900 (2003). 12931177 10.1038/424900a

[b9] Lieberman E., Michel J.-B., Jackson J., Tang T. & Nowak M. A. Quantifying the evolutionary dynamics of language. Nature 449, 713–716 (2007). 17928859 10.1038/nature06137 PMC2460562

[b10] Lambiotte R., Ausloos M. & Thelwall M. Word statistics in Blogs and RSS feeds: Towards empirical universal evidence. J. Informetrics 1, 277 (2007).

[b11] Petersen A. M., Tenenbaum J., Havlin S. & Stanley H. E. Statistical Laws Governing Fluctuations in Word Use from Word Birth to Word Death. Sci. Rep. 2, 313 (2012) 22423321 10.1038/srep00313 PMC3304511

[b12] Gao J., Hu J., Mao X. & Perc M. Culturomics meets random fractal theory: insights into long-range correlations of social and natural phenomena over the past two centuries. J. R. Soc. Interface 9, 1956–1964 (2012). 22337632 10.1098/rsif.2011.0846 PMC3385752

[b13] Zipf G. K. Behavior and the Principal of Least Effort (Addison-Wealey, Cambridge, MA, 1949).

[b14] Heaps H. S. Information Retrieval-Computational and Theoretical Aspects (Academic Press, 1978).

[b15] Kanter I. & Kessler D. A. Markov processes: linguistics and Zipf's law. Phys. Rev. Lett. 74, 4559–4562 (1995). 10058537 10.1103/PhysRevLett.74.4559

[b16] Cancho R. F. i. & Solé R. V. Least effort and the origins of scaling in human language. Proc. Natl. Acad. Sci. U.S.A. 100, 788–791 (2002). 10.1073/pnas.0335980100 PMC298679 12540826

[b17] Gelbukh A. & Sidorov G. Zipf and Heaps Laws' coefficients depend on language. Lect. Notes Comput. Sci. 2004, 332–335 (2001).

[b18] Serrano M. A., Flammini A. & Menczer F. Modeling statistical properties of written text. PLoS ONE 4, e5372 (2009). 19401762 10.1371/journal.pone.0005372 PMC2670513

[b19] Cattuto C., Loreto V. & Pietronero L. Semiotic dynamics and collaborative tagging. Proc. Natl. Acad. Sci. U.S.A. 104, 1461–1464 (2007). 17244704 10.1073/pnas.0610487104 PMC1785269

[b20] Cattuto C., Barrat A., Baldassarri A., Schehr G. & Loreto V. Collective dynamics of social annotation. Proc. Natl. Acad. Sci. U.S.A. 106, 10511–10515 (2009). 19506244 10.1073/pnas.0901136106 PMC2705610

[b21] Zhang Z.-K., Lü L., Liu J.-G. & Zhou T. Empirical analysis on a keyword-based semantic system. Eur. Phys. J. B 66, 557–561 (2008).

[b22] Lansey J. C. & Bukiet B. Internet Search Result Probabilities: Heaps' Law and Word Associativity. J. Quant. Linguistics 16, 40–66 (2009).

[b23] Zhang H.-Y. Discovering power laws in computer programs. Inf. Process. Manage. 45, 477–483 (2009).

[b24] Simon H. A. On a class of skew distribution function. Biometrika 42, 425–440 (1955).

[b25] Simkin M. V. & Roychowdhury V. P. Re-inventing Willis. Phys. Rep. 502, 1–35 (2011).

[b26] Dorogovtsev S. N. & Mendes J. F. F. Languague as an evolving word web. Proc. R. Soc. Lond. B 268, 2603–2606 (2001). 10.1098/rspb.2001.1824 PMC1088922 11749717

[b27] Zanette D. H. & Montemurro M. A. Dynamics of text generation with realistic Zipf's distribution. J. Quant. Linguistics 12, 29–40 (2005).

[b28] Cattuto C., Loreto V. & Servedio V. D. P. A Yule-Simon process with memory. Europhys. Lett. 76, 208–214 (2006).

[b29] Ebeling W. & Pöschel T. Entropy and long-range correlations in literary English. Europhys. Lett. 26, 241–246 (1994).

[b30] Kleinberg J. Bursty and hierarchical structure in streams. Data Min. Knowl. Disc. 7, 373–397 (2003).

[b31] Altmann E. G., Pierrehumbert J. B. & Motter A. E. Beyong word frequency: Bursts, lulls, and scaling in the temporal distributions of words. PLoS ONE 4, e7678 (2009). 19907645 10.1371/journal.pone.0007678 PMC2770836

[b32] Wang D.-H., Li M.-H. & Di Z.-R. Ture reason for Zipf's law in language. Physica A 358, 545–550 (2005).

[b33] Lü L., Zhang Z.-K. & Zhou T. Zipf's law lwads to Heaps' law: Analyzing their relation in finite-size systems. PLoS ONE 5, e14139 (2010). 21152034 10.1371/journal.pone.0014139 PMC2996287

[b34] Eliazar I. The growth statistics of Zipfian ensembles: Beyond Heaps' law. Physica A 390, 3189–3203 (2011).

[b35] Bernhardsson S., da Rocha L. E. C. & Minnhagen P. The meta book and size-dependent properties of written language. New J. Phys. 11, 123015 (2009).

[b36] Goldstein M. L., Morris S. A. & Yen G. G. Problems with fitting to the power-law distribution. Eur. Phys. J. B 41, 255–258 (2004).

[b37] Clauset A., Shalizi C. R. & Newman M. E. J. Power-law distributions in empirical data. SIAM Rev. 51, 661–703 (2009).

[b38] Barabási A.-L. & Albert R. Emergence of scaling in random networks. Science 286, 509–512 (1999). 10521342 10.1126/science.286.5439.509

[b39] Bak P., Tang C. & Wiesenfeld K. Self-organized criticality. Phys. Rev. A 38, 364–374 (1988). 9900174 10.1103/physreva.38.364

[b40] Barabási A.-L. The origin of bursts and heavy tails in human dynamics. Nature 435, 207–211 (2005). 15889093 10.1038/nature03459

[b41] Kim B. J. & Park S. M. Distribution of Korean family names. Physica A 347, 683–694 (2005).

[b42] Pigolotti S., Flammini A., Marsili M. & Martian A. Species lifetime distribution for simple models of ecologies. Proc. Natl. Acad. Sci. U.S.A. 102, 15747–15751 (2005). 16236730 10.1073/pnas.0502648102 PMC1276042

[b43] Dorogovtsev S. N. & Mendes J. F. F. Effect of the accelerating growth of communications networks on their structure. Phys. Rev. E 63, 025101(R) (2001). 10.1103/PhysRevE.63.025101 11308527

[b44] Jeong H., Neda Z. & Barabási A.-L. Measuring preferential attachment for evolving networks. Europhys. Lett. 61, 567–572 (2003).

